# Early Apple Yield Prediction Based on Flowering Stage Image Thinning Simulation Characteristics

**DOI:** 10.3390/plants15071053

**Published:** 2026-03-29

**Authors:** Qihang Yang, Liqun Liu

**Affiliations:** College of Information Science and Technology, Gansu Agricultural University, Lanzhou 730070, China; yangqh@st.gsau.edu.cn

**Keywords:** flowering-stage images, clustering algorithms, blossom thinning simulation, feature optimization, yield prediction

## Abstract

The existing fruit tree yield prediction methods mainly rely on fruit period images or long-term meteorological and soil data, which make it difficult to meet the needs of early yield prediction. In addition, the flowering period images contain complex spatial distribution and severe overlap between flowers, which makes it challenging to directly extract stable structural indicators related to yield. Most existing research has focused on simple statistical indicators such as the number of flowers, while the spatial clustering structure of flowers and their relationship with yield have not been fully explored. Therefore, this article proposes an early apple yield prediction based on flowering stage image thinning simulation characteristics. In this study, blossom images and fruit maturity yield data from 100 apple trees were collected, with flower mask images extracted through standardized image processing. First, the traditional DBSCAN clustering algorithm was enhanced by integrating a KDTree acceleration structure and an adaptive multi-scale mechanism, forming the adaptive multi-scale clustering algorithm (AMS-DBSCAN) to achieve efficient identification of flower clusters and individual flowers. Based on this, two flower thinning simulation strategies based on density and spatial uniformity were designed to model artificial thinning rules and construct multi-dimensional, interpretable phenotypic features. Then, the original statistical features were fused with strategy-generated features and optimized using Lasso. We compared multiple models including XGBoost, BPNN, and SVR for yield prediction. The experimental results showed that XGBoost achieved good predictive performance under the hybrid feature set (R^2^ = 0.856, RMSE = 3.098), which was further improved to R^2^ = 0.900 after feature optimization with Lasso. The results demonstrate that the proposed method enables reliable early yield estimation, providing a new reference for precision management and early decision-making in fruit tree cultivation.

## 1. Introduction

As the world’s largest apple producer, China’s precise and scientific yield forecasts not only guide economic decision-making for fruit farmers but also help regulate market supply and demand and improve the efficiency of the industrial chain [1,2]. Currently, fruit yield prediction primarily relies on joint forecasting methods combining long-term meteorological data with field data or detection methods based on fruiting period imagery [3,4]. However, these methods have limitations such as long time consumption, high cost, or dependence on fruit period data, making it difficult to meet the demand for “early prediction” and “automated processing” in actual production.

In the apple production process, thinning is a key operation that affects fruit quality and yield. Artificial thinning is the basic and most common method, and removing excess flowers through experience can effectively ensure the nutritional supply of fruit trees. However, its labor intensity is high, efficiency is low, and cost is high [5,6]. Chemical thinning is affected by multiple factors such as spraying period, concentration, variety, and environment, resulting in unstable effects, limited fruit tree growth, and potential environmental pollution [7]. Mechanical thinning methods such as handheld striking, airborne vibration, and airborne striking can reduce manual labor and improve efficiency, but they often lead to tree damage, shortened lifespan, and difficulty in accurately controlling the amount of thinning, often resulting in errors or excessive thinning [8].

The flowering period images have a large number of flowers, severe overlap, and complex spatial distribution, making it difficult to directly establish a stable mapping relationship with yield through simple statistics. Only direct and simple information can be obtained through flowering period images or crown top images [9,10]. Lee et al. explored the relationship between yield and flower cluster density through flower cluster detection [11]. Moreira et al. used YOLOv8n to detect and segment flowers and inflorescences, improving the accuracy of early yield prediction [12]. Li Huibin et al. established a single branch yield prediction model based on crown area and fruit quantity, but its application research in yield prediction is still insufficient [4]. The main reasons are that:

(1) In flowering images, the number of flowers is dense and the spatial structure is complex, with common phenomena such as occlusion, overlap, and lighting changes, The existing methods based on object detection or semantic segmentation usually focus on single flower recognition, and their ability to express the overall spatial structure of flowers in complex occlusion scenes is still limited [9,10,11].
(2) The aggregation and dispersion patterns of flowers in images reflect the spatial organization characteristics of inflorescences, but existing research still lacks effective clustering and spatial structure analysis methods for flowering images, making it difficult to stably characterize flower cluster structures at different scales [4,12];
(3) Existing research has mostly focused on low-level visual tasks such as flower detection or semantic segmentation, or has only constructed preliminary yield estimation models based on a single indicator such as the number of flowers. There has been little further exploration of the distribution characteristics of flowers at the spatial structure level and their statistical correlation with yield, and a complete modeling process from the construction of flowering image features to yield prediction has not yet been formed [13,14].

In response to the above issues, this article takes apple flowering period images as the research object and proposes an apple yield prediction method based on the simulation features of thinning flowers in flowering period images. The main innovations include:

(1) The concept of thinning simulation is introduced for the first time during the flowering stage, and a quantifiable “pre thinning post thinning” representation is constructed through image features, providing a new feature generation approach for early yield prediction;
(2) An adaptive scale AMS-DBSCAN clustering algorithm is proposed to identify the structure of flower clusters (high-density distribution areas of flowers in images) at different densities, solving the problem of unstable recognition of multi-scale flower clusters in complex flowering images using traditional methods;
(3) Two thinning simulation strategies are constructed: one based on dynamic retention ratio of flower cluster density, and the other based on individualized retention of spatial uniformity. By quantitatively analyzing the number, area, and spatial uniformity of flower clusters before and after simulation, a multi-level phenotype feature set of flowering period is formed, providing data support for early yield statistical prediction based on image features.

In summary, this article takes apple flowering period images as the research object, constructs interpretable flower spatial structure characteristics through a thinning simulation process, and based on this, achieves early prediction of apple single plant yield.

## 2. Materials and Methods

### 2.1. Dataset Introduction

The dataset was collected in mid-April 2025, using standard smartphones as the imaging equipment, with the primary collection site located in the core apple cultivation area of Tianshui City, Gansu Province, China. The dataset was collected from the same orchard, and all fruit trees were planted under similar management conditions.

To achieve yield prediction during the flowering stage, it is necessary to collect image samples of varying flower quantities and distribution states. Therefore, selecting different time periods (morning, noon, evening) under both forward and backward lighting conditions, and covering a variety of shooting angles (frontal, top-down 30°, bottom-up 30°) and background scenes (sky, buildings, distant fruit trees, etc.) were necessary to enhance sample diversity and model generalization capability, providing representative flowering-stage samples for subsequent spatial distribution modeling and yield prediction. A total of 141 high-quality original images were obtained during the entire collection process. After screening, 100 images covering the blooming state of the entire apple tree from 100 different sample apple trees were retained, and the image size was uniformly adjusted to 3072 × 4096 pixels.

**Figure 1:**
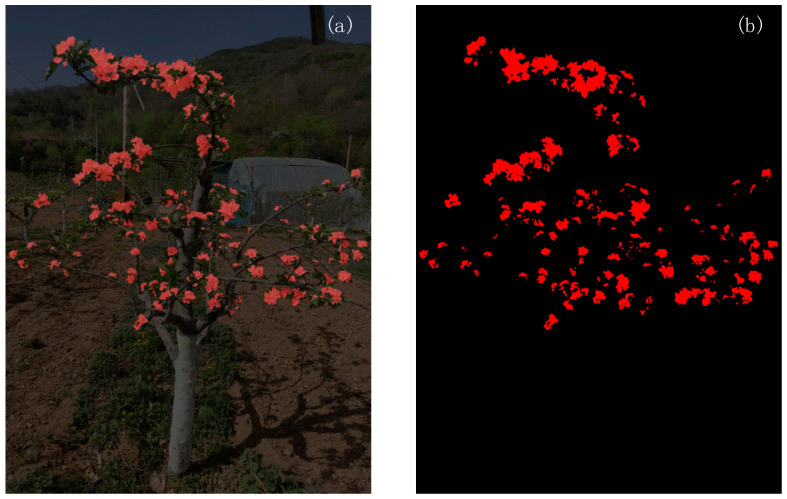
Dataset schematic diagram. (**a**) Schematic diagram of apple flower extraction area; (**b**) Schematic diagram of mask for apple flower area.

To support the application of apple flowering period images in thinning simulation and yield prediction, this study used a sliding window cropping strategy on the collected dataset, with a step size of 512 pixels, to divide the original image into multiple small images of 1024 × 1024 pixels. After accumulating 4800 sample images, Labelme was used for unified labeling, and the training and testing sets were defined at an 8:2 ratio. After 100 epochs of training using Deeplabv3+, the model achieved a mean Intersection over Union (mIoU) of 92.92%, a mean Precision (mRecall), a mean Recall (mRecall), and a mean F1 score (mF1) of 96.64%, 95.86%, and 96.24%, respectively. All experiments were conducted in Python (version 3.8) using PyTorch (version 2.4) with CUDA 12.4 acceleration, and the code was developed in PyCharm (version 2024). DeepLabv3+ was used to separate the target flower object from the background and create corresponding flower mask images as dataset samples. The content of the image dataset is shown in Figure 1.

**Figure 2:**
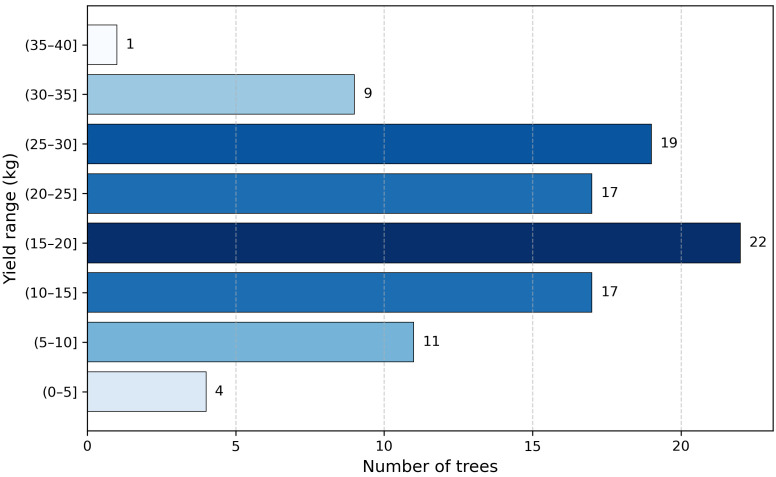
Production distribution histogram.

In this study, 100 apple fruit tree samples were collected during the flowering period as research objects. After image acquisition, experienced fruit farmers carried out normal thinning and fruit thinning operations on the fruit trees, and manually picked the apple fruit trees during the fruit ripening period. Electronic weighing devices (SENSSUN, Zhongshan, Guangdong, China) were used to weigh and record the yield data of apples harvested from 100 fruit trees (excluding samples of naturally fallen apples during growth). The yield data of each tree is retained in kilograms, with a sample yield range of 4.6–35.9 kg, with an average yield of 18.45 kg. The above sampling method ensures that the yield distribution has a certain representativeness, which facilitates the establishment of a mapping relationship between flowering characteristics and final yield. The yield distribution information is shown in Figure 2.

### 2.2. Flower Cluster Recognition and Clustering Methods

**Figure 3:**
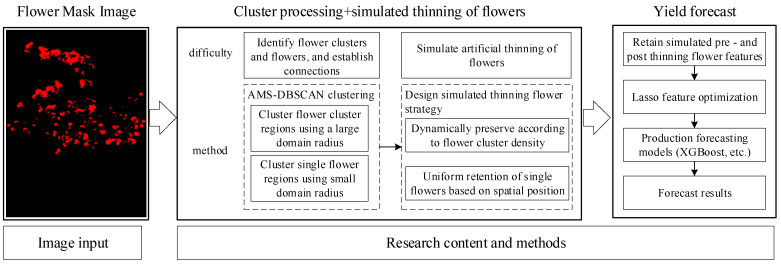
Overall technical roadmap.

After completing the collection of sample data, the mask image of apple fruit tree flowering period only separates flowers from complex environments, and the original feature information (total number of flowers, overall area) is difficult to express as spatial distribution and cluster structure size information. Further spatial structure analysis of flowers is needed to extract more representative phenotypic features of the flowering period, providing a reliable data basis for subsequent thinning simulation and yield prediction. This method takes flower segmentation results as input and constructs a complete analysis framework from flowering period images to yield estimation through four main steps: flower cluster clustering, thinning simulation, feature construction, and yield prediction. The overall technical roadmap is shown in Figure 3.

Firstly, the flower mask image obtained by DeepLabv3+ is used as input data to extract the spatial coordinates of flower pixels, and the flower cluster structure is identified through density clustering algorithm. Considering the significant differences in scale and density of apple flower clusters, traditional fixed parameter clustering algorithms are difficult to adapt to complex flowering period distribution characteristics. Therefore, this paper introduces an adaptive scale adjustment mechanism and KDTree acceleration structure based on the DBSCAN algorithm, and constructs an adaptive multi-scale density clustering algorithm (AMS-DBSCAN) to effectively identify single flowers and flower cluster structures.

After obtaining information on the structure of flower clusters, this article further combines the experience of artificial thinning flower in orchards to design two thinning simulation strategies: one is a dynamic retention strategy based on flower cluster density, which simulates the thinning principle of “dense is sparse” by moderately reducing high-density flower clusters; the second is a uniform thinning strategy for single flower space which controls the spatial spacing between flowers to flowers to make the preservation of flowers more evenly distributed in space. By simulating these two strategies, multidimensional features such as the number of flower clusters after thinning, retention area, and spatial uniformity can be generated.

Finally, the statistical features of the original flower clusters are fused with the features generated by two thinning strategies to construct a multidimensional phenotype feature set of flowering images. The Lasso method is used to screen and optimize the features to reduce redundant information and improve the model’s generalization ability. Finally, the optimized features are input into multiple machine learning models (such as XGBoost, BPNN, and SVR) for training and comparison, establishing a mapping relationship between flowering image features and actual fruit yield during the ripening period, and achieving early prediction of apple yield.

### 2.3. Improved Clustering Algorithm AMS-DBSCAN

#### 2.3.1. AMS-DBSCAN

**Figure 4:**
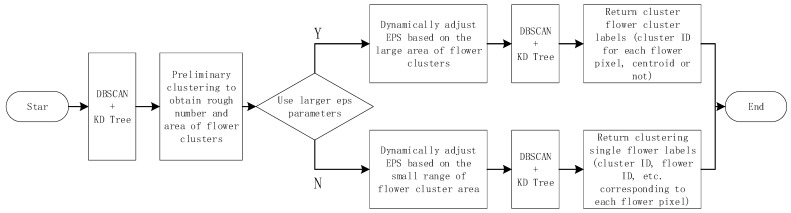
AMS-DBSCAN flowchart.

In the process of this study, due to the irregular characteristics of the shooting perspective and the target object itself, there were significant differences in the size, shape, and density distribution of flower clusters in the raw data images obtained. The traditional DBSCAN algorithm uses a fixed neighborhood radius eps, which makes it difficult to effectively identify sparse independent flowers and dense large flower clusters, and can easily lead to single flowers being misjudged as noise. When traditional algorithms process data samples, each data point requires multiple neighborhood queries, and the algorithm complexity is O(n^2^), resulting in lower processing efficiency in large-scale image scenes [15]. In response to the above issues, this study proposes an adaptive multi-scale DBSCAN clustering method (AMS-DBSCAN), and the implementation flowchart of the method is shown in Figure 4.

On the basis of maintaining the DBSCAN algorithm, this framework introduces an area adaptive adjustment mechanism and KDTree acceleration strategy to improve the adaptability and computational efficiency of clustering algorithms in flowering mask images, providing a reliable flower cluster structure foundation for subsequent thinning simulation tasks. The algorithm first fixed the neighborhood radius EPS to 10 for clustering, obtaining information on the area and number of flower clusters. Due to significant differences in spatial distribution characteristics between isolated flowers and dense flower clusters, single layer clustering and cluster layer clustering were distinguished by the size set by EPS. In areas with dense flower distribution, a smaller neighborhood radius (EPS) is required to prevent adjacent flowers from excessively merging into a cluster. On the contrary, the EPS in the flower cluster area can be set to larger values to reduce the non-aggregation of flower clusters caused by small variations in EPS. Therefore, the proposed method introduces a conditional branching mechanism that dynamically adjusts clustering parameters based on estimated clustering features. This adaptive strategy improves clustering stability in different scenarios and provides more reliable inputs for subsequent processes.

#### 2.3.2. Definition of Feature Space

In order to identify spatially clustered flower clusters in flowering images, the AMS-DBSCAN algorithm performs clustering in a two-dimensional pixel coordinate space. The flower region mask obtained by semantic segmentation is M(x,y), and its non-zero pixels represent all regions of the flower. For each input image, the spatial coordinate information of all flower pixels is first extracted using the following method:

(1) $${Flower}_{coords}=\left\{\left(x_{i},y_{i}\right)|M\left(x_{i},y_{i}\right)\neq 0\right\},\;\;i=1,2,\ldots ,N$$

where N is the total number of flower pixels, $x_{i}$ and $y_{i}$ represent the column coordinates and row values of pixel points in the image, respectively. After extracting the pixel coordinates of all flowers, a two-dimensional feature matrix can be obtained:

(2) $$P=\left[\begin{array}{cc} y_{1} & x_{1}\\ \ldots & \ldots \\ y_{N} & x_{N} \end{array}\right]\in R^{N\times 2}$$

This matrix constitutes the feature space of AMS-DBSCAN clustering. The clustering process adopts the Euclidean distance metric and uses spatial proximity as the criterion for clustering combination. The formula is as follows:

(3) $$d_{ij}=\sqrt{{\left(x_{i}-x_{j}\right)}^{2}+{\left(y_{i}-y_{j}\right)}^{2}}$$

In this study, due to the obvious clustering distribution pattern of flowers in their natural state, adjacent pixels on the image plane also correspond to adjacent positions in real space. Therefore, clustering results based on two-dimensional coordinates can effectively describe the spatial organization structure of flower clusters. The clustering process is carried out in a two-dimensional pixel coordinate space, which can directly reflect the spatial distribution characteristics of flowers in the image, and has good physical consistency and interpretability. In addition, the distance calculation process in two-dimensional Euclidean space is concise and can be efficiently combined with the KDTree index structure, significantly improving the algorithm’s operational efficiency while ensuring clustering accuracy.

#### 2.3.3. Double Layer Adaptive EPS Regulation Mechanism

The fixed EPS parameters in traditional DBSCAN are difficult to adapt to image regions of different sizes. This study proposes an adaptive neighborhood radius adjustment mechanism. The core idea of this mechanism is that the area of the clustering region is positively correlated with the required neighborhood radius, that is, the larger the region area, the larger the required EPS value, in order to avoid misclassifying a large cluster into multiple small clusters.

(1) Macro-clustering: The purpose of this level is to aggregate adjacent flower pixels in spatial position into individual flower clusters. In order to address the significant differences in flower cluster size among different images, this study designed a dynamic adaptive EPS mechanism for this level. The core idea is that as the area of a flower cluster increases, the required neighborhood radius should also increase accordingly to avoid misclassifying a large cluster into multiple small clusters. The calculation formula for adaptive EPS is as follows:
  (4) $$R=\frac{{Area}_{cluster}}{{Area}_{target}}$$
  (5) $${eps}_{cluster}={eps}_{base}+\beta \cdot \ln\left(R\right)$$

Among them, ${Area}_{cluster}$ is the total area of the current flower area to be clustered, ${Area}_{target}$ determines the area threshold of the flower cluster by analyzing the area distribution of the flower cluster samples, R is the area ratio coefficient, ${eps}_{base}$ is the base radius, and β is the adjustment coefficient used to control the strength of the influence of area on eps. The ${eps}_{cluster}$ will ultimately be constrained within a preset interval to ensure the stability of the algorithm’s parameters. This function has the ability to suppress parameters in areas with extremely large flower clusters by adding logarithms during the design process, so that the eps value slowly increases with the increase of area, preventing parameter explosion, and the range of parameter values is shown in Table 1.

(2) Micro-clustering: After identifying the region of each flower cluster, fine-grained clustering is performed within the flower cluster to separate the individual flowers that exist. Therefore, the variation range of the ${eps}_{cluster}$ is relatively small, and when fine tuned based on the total area of the flower cluster during the individual flower clustering differentiation process, its variation range is much smaller than that of the flower cluster hierarchy. This ensures the sensitivity and consistency of the algorithm in handling local details.

By combining flower clusters with microscopic single flowers in a two-level design, the AMS-DBSCAN algorithm can effectively solve the problem of clustering failure in traditional algorithms in scenarios with large scale differences. It retains the overall shape of flower clusters while capturing individual flowers, providing diverse and comprehensive feature information for subsequent yield prediction research.

#### 2.3.4. Double Level Clustering Architecture Based on KDTree Acceleration

KDTree is a spatial partitioning structure based on binary trees, which recursively divides the dataset into hyper rectangular regions according to different dimensions, thereby achieving efficient indexing of point sets [16]. When performing neighborhood search, KDTree can quickly eliminate areas that are unlikely to have neighbors and continue searching only in nodes that intersect with the query radius. Compared to the O(n) complexity of brute force search, the average neighborhood access time of KDTree only requires O(logn), Using this structure can effectively reduce the overall complexity of DBSCAN, with a time complexity of O(nlogn). Therefore, this article introduces the KDTree data structure in the clustering algorithm process, and inputs the two-dimensional coordinate set $P=\left\{\left(y_{i},x_{i}\right)\right\}$ of flower pixels to achieve fast retrieval of adjacent pixel sets within a specified radius.

In this study, the flower pixels first need to be clustered throughout the entire image to identify adjacent flower clusters. The amount of data at this stage is huge, and if traditional neighborhood search methods are used, the computational cost is extremely high. The application of KDTree can quickly complete large-scale nearest neighbor retrieval, effectively supporting dynamic adjustment of adaptive ε on clusters of different areas, thereby ensuring clustering efficiency. After obtaining the rough distribution data of flower clusters through coarse clustering, fine-grained single flower clustering is performed. Although the point set size at this stage is relatively small, due to the higher requirements for accuracy and sensitivity of neighborhood search in single flower clustering, the efficient indexing of KDTree can still improve the running speed while maintaining high-resolution recognition, ensuring that the algorithm can maintain stable efficiency in batch image processing tasks. The average running time of the AMS-DBSCAN algorithm accelerated by KDTree is only about 30–40% of that of traditional DBSCAN. As the data volume increases, its efficiency advantage becomes more apparent. By combining a two-level adaptive parameter mechanism, this optimization ensures that the algorithm has stable clustering accuracy and meets the requirements of real-time and scalability in large-scale image processing scenarios.

### 2.4. Flower Thinning Strategy

Under the condition of a single flowering period image, some information related to the physiological status of flowers and the carrying capacity of branches is difficult to directly obtain, so visual methods are difficult to fully reproduce the above experience. However, the spatial distribution characteristics, local density, and the morphology and area of individual flowers in flower clusters have good observability, which can to some extent reflect the spatial competition between flowers.

Based on the above modeling information, after completing the dual level clustering of AMS-DBSCAN algorithm, this paper obtained the flower cluster distribution structure and single flower segmentation results of the apple tree flowering period. Further combining the clustering results of flower clusters with individual flower characteristics, two types of thinning simulation strategies were designed from the perspectives of flower cluster density control and single flower quantity control, to approximate some empirical principles in artificial thinning at the visual level. The following thinning simulation methods have not yet replaced thinning operations in real orchards.

(1) Dynamic retention strategy for flower cluster density: Flower cluster density reflects the degree of aggregation of flowers in local space, and overly dense flower clusters can lead to competition for nutrients in subsequent fruits and affect fruit quality. Therefore, this article designs a density constrained thinning simulation preservation function based on the characteristics of flower cluster area and spatial density under visually observable conditions, to approximate the empirical principle of “density must be sparse” in artificial thinning:
  (6) $$r=clip\left(r_{base}\cdot A_{single}\cdot A_{round}\cdot A_{compact},\;r_{min},\;r_{max}\right)$$

Among them, $r_{base}$ is divided into multiple retention ratios based on the area of the flower cluster (the larger the area, the lower the retention ratio); $A_{single}$ is the number of single flowers in the current flower cluster, and the more single flowers there are, the lower the retention rate; $A_{round}$ evaluates shape and regularity by calculating the roundness of clusters, and clusters with regular shapes have an appropriate increase in retention rate. There is a negative correlation between $A_{compact}$ and cluster compactness, and clusters with high compactness will have a slightly reduced retention rate. Clip is used to constrain the retention rate range of flower cluster area, controlled within $\left[r_{min},r_{max}\right]$, and the range of parameter values is shown in Table 2.

**Figure 5:**
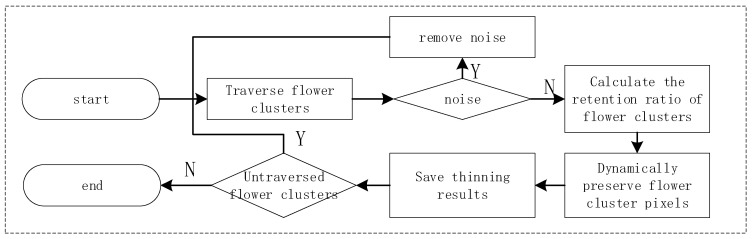
Flowchart of dynamic retention strategy for flower cluster density.

Dynamic retention strategy for flower cluster density: The implementation process is shown in Figure 5. The algorithm first traverses the area of each flower cluster based on the clustering results. For clusters with smaller areas, the retention rate is set to 0 and treated as noise to be directly removed. Then, the above function is used to obtain the retention ratio of each cluster, with priority given to retaining pixels near the center of the cluster, forming a representative spatial structure of flower density.

**Figure 6:**
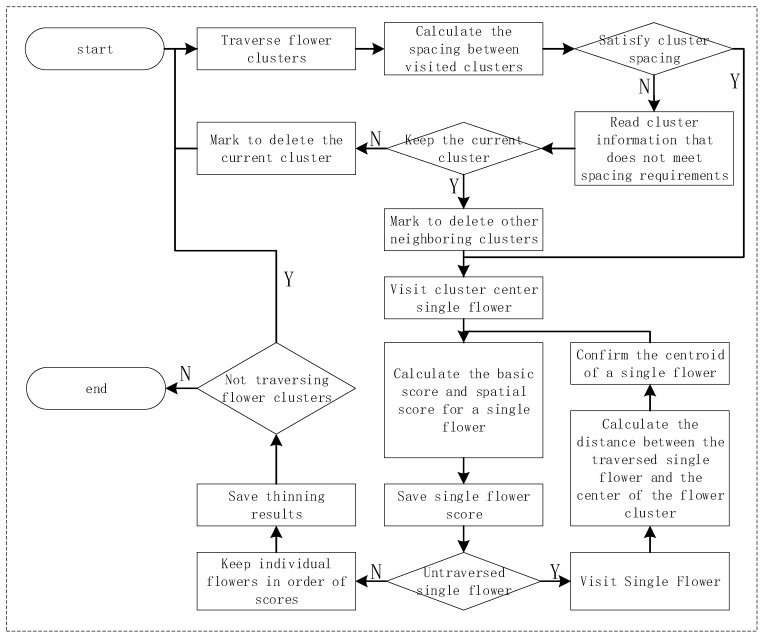
Flowchart of uniform thinning strategy for single flower space.

(2) Uniform thinning strategy for single flower space: After obtaining the preliminary parameters and data of the flower cluster, strategy two tends to retain specific flowers, control the number of flowers while maintaining a more uniform distribution of flowers within the cluster, and avoid situations such as local overcrowding or leaning towards the edges, and the thinning flower process is shown in Figure 6.

Its core idea is that before the thinning of flowers within the cluster begins, in order to avoid the phenomenon of local overcrowding caused by the distance between flower clusters being only greater than the neighborhood radius, the distance between clusters is first filtered. If the distance between the centroids of two clusters is less than the threshold, only the flower cluster with the larger area will be retained to avoid redundancy caused by overly fine cluster segmentation, thereby enhancing the stability of the thinning strategy. Under the constraint of target quantity T (positively correlated with cluster size), the flowers to be retained are selected by visiting each flower in the cluster one by one and calculating their basic and spatial scores. The calculation formula is as follows:

(7) $$S_{total}=S_{base}\cdot S_{spatial}$$

Among them, $S_{base}$ is related to local features such as flower area and roundness; $S_{spatial}$ is used to balance the two requirements of “center first” and “uniform coverage”. In the same flower cluster, each single flower small cluster is traversed sequentially. In the initial stage, the single flower small cluster located at the center of the large cluster is prioritized. In the subsequent selection process, the average distance between the determined small clusters is taken into account while considering the distance from the cluster center, so as to ensure that the single flower small clusters are spatially dispersed when selecting the core point and avoid excessive concentration in local areas.

### 2.5. Production Forecasting Model

Due to the high complexity of the mapping relationship between flower spatial distribution structure, quantitative statistical characteristics and final yield, it is difficult for a single model to simultaneously depict the statistical correlation between multi-scale spatial characteristics and yield response. Stepwise multiple regression [17] (SMR) is chosen to establish a linear model, which can gradually screen out the main influencing factors in the case of a large number of features and construct a concise prediction equation. Decision tree-based algorithms are introduced, including Random Forest [18], XGBoost [19] and LightGBM [20]. Random Forest utilizes a combination of multiple decision trees to improve the robustness of the model, XGBoost improves generalization ability through iterative optimization and regularization design, while LightGBM uses an efficient histogram splitting method to significantly shorten training time while maintaining accuracy. SVR [21] is based on computational learning theory and uses kernel functions to map data to high-dimensional space to capture complex nonlinear relationships. Although it has a high computational cost, it is suitable for prediction problems with limited sample sizes and high feature dimensions. MLP [22] and BPNN [23] have also been incorporated into deep learning models, which can automatically learn potential relationships from input features and have strong fitting ability for complex nonlinear mappings. This study considered multiple approaches in model selection, including linear and nonlinear, statistical regression and ensemble learning, shallow methods and deep networks, aiming to reveal the relationship between flowering phenotype characteristics and yield from different perspectives.

### 2.6. Evaluation Metric

For a comprehensive performance evaluation of various yield prediction models, four metrics are employed in this paper: coefficient of determination (*R*^2^), root-mean-square error (RMSE), mean absolute error (MAE), and relative error (RE). The coefficient of determination (*R*^2^) is used to quantify the goodness of fit between the model predictions and the ground-truth values. The root-mean-square error (RMSE) reflects the overall deviation between predicted values and ground-truth values. The mean absolute error (MAE) is adopted to represent the average absolute difference between predicted values and ground-truth values. The relative error (RE) measures the proportional deviation of predicted values relative to the ground-truth values. Their calculation formulas are given in Equations (8)–(11).

(8) $$R^{2}=1-\frac{\sum_{i-1}^{n}{\left(y_{i}-{\hat{y}}_{i}\right)}^{2}}{\sum_{i-1}^{n}{\left(y_{i}-{\bar{y}}_{i}\right)}^{2}}$$

(9) $$RMSE=\sqrt{\frac{1}{n}\sum_{i=1}^{n}{\left(y_{i}-{\hat{y}}_{i}\right)}^{2}}$$

(10) $$MAE=\frac{1}{n}\sum_{i=1}^{n}\left|y_{i}-{\hat{y}}_{i}\right|$$

(11) $$RE=\frac{1}{n}\sum_{i=1}^{n}\frac{\left|y_{i}-{\hat{y}}_{i}\right|}{\left|y_{i}\right|}$$

Among them, $y_{i}$ represents the true value of the i-th sample, ${\hat{y}}_{i}$ indicates the predicted result, ${\bar{y}}_{i}$ denotes the sample mean, and n is the total number of samples.

*R*^2^, RMSE, and MAE are widely used in crop yield prediction research [24]. RE is introduced to normalize the prediction error relative to the actual yield value, allowing for a more intuitive comparison across different yield ranges. These indicators are used to comprehensively evaluate the accuracy and reliability of the model.

## 3. Results

### 3.1. Comparative Experiment

#### 3.1.1. Visualization Results of Thinning Flowers

**Figure 7:**
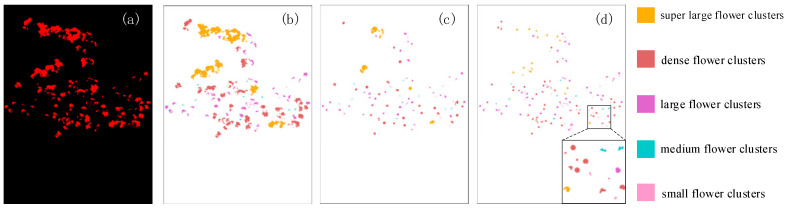
Sparse flower simulation diagram. (**a**) Original image; (**b**) Clustering results of flower clusters; (**c**) The results of the dynamic retention strategy for flower cluster density; (**d**) The results of the uniform thinning strategy for single flower space.

To visually demonstrate the differences in spatial structure between two thinning strategies and their impact on the preservation status of flower clusters, this paper presents a visualization of the simulated thinning results, as shown in Figure 7. In order to facilitate the observation of the retention of flower clusters of different scales during the simulation process, graded colors are used to indicate the size of flower clusters in the figure. By comparison, it can be seen that there are significant differences in the preservation methods of the two strategies on flower clusters of different scales, reflecting the differential characteristics of spatial structure reconstruction under different thinning principles.

To further verify the rationality of the constructed thinning simulation strategy in intensity control, this paper calculated the average retention ratio of pixels in the flower area after thinning under two strategies. The results showed that the average retention ratios of dynamic retention strategy for flower cluster density and uniform thinning strategy for single flower space were 27.5% and 19.2%, respectively. In actual production, the thinning intensity of apples is usually affected by factors such as variety, tree vigor, and target yield. The proportion of flower retention is generally within the empirical range of 20–40%, indicating that the proposed thinning strategy has certain practical rationality in thinning intensity.

#### 3.1.2. Feature Grouping

In order to extract phenotype features related to yield from apple flowering images, multi-level flowering features were extracted from spatial distribution, morphological structure, and thinning strategy simulation outputs by clustering single flowering apple trees. According to the phenotypic characteristics of the flowering period, it can be divided into seven categories, and the main characteristic types and explanations are shown in Table 3.

This study extracted a total of 50 quantitative features, covering the quantity, morphology, and spatial distribution characteristics of tree flowering phenotypes. To compare the impact of different feature sources on yield prediction performance and facilitate subsequent feature selection and model training, this paper divides the extracted features into the following five feature groups:

(1) Original feature set: preserves 23 features extracted from the original image, including the number of flower clusters, total number of single flowers, average roundness and compactness, variance of flower cluster area, and dispersion of single flower distribution.
(2) Strategy1 feature set: includes 13 features such as the number of flower clusters, reserved area, and average dynamic retention ratio preserved under the density feature of thinning strategy one.
(3) Strategy2 feature set: includes 14 features in total, including the number of flowers and flower clusters preserved for spatial uniformity in the thinning strategy 2, the preserved flower area, and cluster features of different cluster levels.
(4) Mixed_strategy feature set: composed of strategy1 and strategy2 features, totaling 27 features.
(5) Mixed_all feature set: consisting of original, strategy1, and strategy2 features, totaling 50 features.

#### 3.1.3. Feature Optimization and Screening

After completing the feature extraction of flowering period images, a multidimensional feature set is obtained, including flower quantity features, spatial distribution features, and thinning simulation features. However, there may be strong correlation or redundant information between some features. If all features are directly input into the prediction model, it will not only increase the complexity of the model, but also lead to overfitting, thereby affecting the generalization ability of the model. Therefore, this article adopts Lasso regularization method to screen and optimize features. Lasso regression introduces the L1 norm penalty term into the traditional linear regression loss function, and achieves feature selection through sparse constraints on regression coefficients. The optimized loss wave function can be expressed as:

(12) $$\sum_{i=1}^{n}{\left(y_{i}-{\hat{y}}_{i}\right)}^{2}+\alpha \sum_{j=1}^{p}\left|\beta_{j}\right|$$

Among them, $y_{i}$ is the true value of the i, ${\hat{y}}_{i}$ is the predicted result, $\beta_{j}$ is the coefficient of the j-th feature, p is the number of independent variables, and the degree of contraction of the regression coefficient is controlled by adjusting α.

This study first standardized all features using Z-score, and then used Lasso regression (with a fixed regularization parameter of α = 0.01) to select features from each original feature group. After the model converges, all features with regression coefficients $\left|\beta_{j}\right|>0$ are considered to contribute to the yield prediction results. The feature importance is sorted based on the absolute value of the regression coefficient, and the top 8 features with the highest contribution are retained as the optimized feature subset. The optimized feature groups are named: Original_opt, strategy1_opt, strategy2_opt, mixed_strategy_opt, and mixed_all_opt. Taking mixed_all_opt as an example, the feature situation is shown in Table 4. Compared with mixed-all, the dimension of the mixed_all_opt feature group is significantly reduced, and the main structural and spatial information is still retained.

### 3.2. Prediction Results of Yield per Individual Apple Tree

To evaluate the application effectiveness of different feature groups constructed from flowering period images in predicting the yield of individual fruit trees, this study conducted a systematic comparative analysis of various commonly used regression models. The selected models include: XGBoost [19], MLP [22], BPNN [23], RF [18], SMR [17], SVR [21] and LGBM [20]. The mixed feature set mixed_all consisting of the original features, strategy one, and strategy two was selected as the input content.

Due to the fact that the experimental sample size only includes 100 records, in order to reduce the impact of randomness caused by the limited dataset size, this study adopted a fivefold cross validation strategy. The dataset was randomly divided into five subsets, with four subsets used for training and one subset used for testing. This process was repeated five times to ensure that each subset was only used for testing once, and the average result was used as the final model’s various indicator results. The *R*^2^, RMSE, MAE, and RE indicators of each model in the test set were calculated separately. The experimental results are shown in Table 5 and Table 6.

The results indicate that most models on the training set can achieve high fitting accuracy on the unoptimized feature set, with the XGBoost model achieving the best performance and the highest *R*^2^ on its test set, demonstrating strong fitting ability. However, in the test set results, some models showed a significant decrease in predictive performance, indicating that directly using all features for modeling can easily lead to overfitting problems. After introducing Lasso feature optimization, the overall predictive performance of each model on the test set was improved. The *R*^2^ of the XGBoost model on the optimized feature set was increased to 0.900, while the RMSE and MAE both decreased significantly, indicating that feature optimization can effectively improve the generalization ability of the model. The SMR model also achieved good prediction performance after optimizing features, with an *R*^2^ of 0.907 in the test set. From the comprehensive results of the training and testing sets, it can be seen that Lasso feature optimization can effectively reduce the interference caused by redundant features and improve the predictive stability of the model.

### 3.3. Analysis of the Contribution of Flower Thinning Simulation Characteristics to Yield Prediction

On the basis of determining XGBoost as the model with better comprehensive performance in the previous experiment, a comparative analysis was conducted for different feature combinations to further analyze the practical role of the thinning simulation strategy proposed in this study in yield prediction. The XGBoost model was used for each feature group and evaluated through fivefold cross validation. The experimental results are shown in Table 7 and Table 8.

The experimental results show that the original feature set only includes basic statistical features such as flower quantity and area. The *R*^2^ of the test set is 0.832, indicating relatively limited predictive ability. This suggests that relying solely on the statistical features of the original flowering period is difficult to fully characterize complex yield variation patterns. The *R*^2^ of the strategy1 feature set on the test set is 0.797, indicating that the predictive performance has not been significantly improved. This suggests that some of the sparse simulation features generated by this strategy have some noise, which interferes with the model prediction. In contrast, the *R*^2^ of the strategy2 feature set test set increased to 0.875 and the RMSE decreased to 2.888 kg, indicating that the thinning simulation strategy based on spatial uniformity can more effectively reflect the relationship between flower distribution structure and yield.

After further integrating the features of the two thinning strategies, the *R*^2^ of the mixed_strategy feature group on the test set increased to 0.856, and the overall predictive performance was better than that of a single strategy feature, but there was still some redundant information. After optimizing the features using the Lasso method, the overall predictive performance of each feature group on the test set was improved. Among them, the mixed’all_opt feature group performed the best, with an R^2^ of 0.900 on the test set and RMSE and MAE reduced to 2.581 kg and 2.043 kg, respectively. The results showed that by integrating multi-source flowering period features and combining feature optimization to screen key variables, the impact of redundant information on model training can be effectively reduced, thereby significantly improving the generalization ability and prediction accuracy of the apple yield prediction model.

## 4. Discussion

### 4.1. Data Limitations

The purpose of this study was to evaluate the predictive potential of flowering period image features for early yield estimation, rather than modeling all agronomic factors that affect yield. Therefore, the proposed model mainly focuses on exploring the relationship between flowering period characteristics and apple yield. Although factors such as tree age, rootstock type, canopy structure, and irrigation system may affect the final yield, these variables were not taken into account in the dataset collected for this study.

### 4.2. Limitations of AMS-DBSCAN Clustering

The clustering results generated by AMS-DBSCAN in this study were not directly validated using manually annotated ground truth values of flower clusters. On the contrary, the effectiveness of clustering results is indirectly evaluated through yield prediction performance. Future work will focus on establishing a manually annotated flower cluster dataset to quantitatively evaluate clustering accuracy.

### 4.3. Analysis of the Ability to Characterize Yield Based on the Structural Characteristics of Flowering Period

From the perspective of agricultural production, a reasonable inflorescence structure and spatial distribution can reduce nutrient competition, improve lighting conditions, and thus affect fruit setting rate and fruit development quality. The results of this study indicate that relying solely on basic statistical features such as flower quantity and area for yield prediction is difficult to fully reflect the true load structure of apple trees during the flowering stage. The predictive ability of the original feature set is relatively limited. Combining the feature set constructed by thinning simulation, the model’s prediction accuracy is significantly improved, indicating that the spatial structure characteristics of flowering period have important representational significance in the process of yield formation.

### 4.4. The Impact of Different Thinning Simulation Strategies on Yield Prediction

Among the two thinning flowers simulation strategies, the uniform thinning strategy for single flower space has a more significant effect on improving yield prediction. The uniform distribution of flowers in space during the flowering stage may better reflect the potential yield structure of fruit trees than simple density control. From practical production experience, thinning flowers not only emphasizes the principle of “density must be sparse”, but also emphasizes reasonable spatial distribution and load balancing. The results of this study are consistent with this production law, indicating that quantitative analysis of spatial structure through images has certain agricultural rationality. It should be noted that the thinning strategy proposed in this study cannot currently replace manual thinning flowers operations in real orchards. Its purpose is to provide quantifiable feature construction methods for early yield prediction.

### 4.5. Impact of Feature Fusion and Feature Screening on Model Stability

The research results show that under the condition of multi-source feature fusion, the prediction accuracy is further improved, and after feature screening through Lasso method, the model performance reaches the best state. This indicates that when predicting yield during the flowering stage, it is more crucial to construct structural features reasonably and remove redundant variables than simply increasing model complexity.

### 4.6. Future Development Direction

This study provides a new research approach for early decision-making of fruit trees based on image information, but there is still room for improvement. Future work can be carried out in the following aspects: firstly, exploring instance level flower or inflorescence modeling methods to make flower thinning simulations more realistic; secondly, combining multi temporal or multimodal information to characterize the differences in flower growth quality; thirdly, expanding multiyear and multi-regional data to verify the generalization ability of the model.

## 5. Conclusions

This paper proposes an early yield prediction method based on the structural features of flowering period images to address the problem of excessive reliance on fruit period images and long-term meteorological data for apple yield prediction, making it difficult to achieve early assessment. In this study, images of 100 apple trees during the flowering period and their corresponding mature yield data were collected. Structural information was extracted during the flowering period, and a statistical correlation between flowering phenotype characteristics and final yield was established. The following tasks were completed:

(1) Feature extraction of flowering images: Cluster analysis was used to identify and statistically analyze the structure of flowers and flower clusters, and two types of simulated thinning strategies were constructed based on this. One is the dynamic retention strategy for flower cluster density, which reflects the empirical principle of “density must be sparse” in artificial thinning; the second is a uniform thinning strategy for single flower space, which reconstructs the spatial structure of the flowering period by adjusting the spacing between flowers. The above method achieves quantitative expression of flowering load structure within the scope of visual modeling, providing biologically significant structural indicators for subsequent yield prediction.
(2) Yield prediction: Integrating the original statistical features of flowering period with the structural features generated by simulated thinning, and optimizing variable combinations through feature screening methods to construct a multi model comparative analysis framework. The results indicate that the model’s prediction accuracy is significantly improved after fusing structural features, with an *R*^2^ of 0.900. The ablation analysis further indicates that spatial uniformity related features have important contributions to the prediction effect, indicating that the spatial structure of flowering period has an indicative role in the formation of final yield.

In summary, this article provides a feasible path for early apple yield prediction based on flowering period images. In the future, by combining multiyear data and physiological indicators, the generalization ability of the thinning simulation mechanism and prediction model can be further improved.

## Figures and Tables

**Table 1:** Introduction to parameter information.

Parameter Name	Reference Value Range	Parameter Description
${Area}_{cluster}$	\	No fixed range, the total area of the pixel region to be sampled.
${Area}_{target}$	1000~1500	Based on the statistical distribution of the sample area in the flower region, the benchmark threshold is determined, with a value of 1200 in this article, corresponding to approximately 60% percentile of the cumulative distribution of the samples.
R	1~100	The area ratio coefficient constrains the range of values to avoid extreme values.
${eps}_{base}$	5~15	The basic neighborhood requirements for range adaptation of clustering at different scales are as follows: flower cluster base = 10 and single flower base = 7 in this article.
β	1.5~2	It is necessary to ensure that EPS grows slowly with the area. If *β* is too large, it will cause parameter explosion, and if it is too small, the adaptive effect will be weak. This article takes a value of 1.8.
${eps}_{cluster}$	\	No fixed range. The final EPS value for clustering is set to a maximum value of 20 to ensure parameter stability.

**Table 2:** Introduction to parameter information.

Parameter Name	Reference Value Range	Parameter Description
$r_{base}$	Graded value	Based on the area of the flower cluster, the target single flower area (1200 pixels^2^) was referenced. Extremely small areas with an area of less than 300 pixels^2^ are not retained; <500 fully reserved; <1000 reserve 0.7; <5000 keep 0.5; <10,000 keep 0.3; reserve 0.2 for ≥10,000.
$A_{single}$	0~0.3	The single flower count adjusts the retention ratio downward via the formula 1 min (single flower count/20, 0.3), with a maximum downward adjustment of 30%.
$A_{round}$	1~100	The circularity adjusts the retention ratio upward via the formula 1 + min (2 × circularity, 0.2), with a maximum upward adjustment of 20%. The closer the circularity is to 1 (the more circular the flower), the higher the retention ratio.
$A_{compact}$	0~0.2	The compactness adjusts the retention ratio downward via the formula 1 − 0.2 × min(compactness/50,000, 1). The normalization constant of 50,000 is derived from the median of typical compactness values in the pre-experiment, ensuring the correction amplitude is controlled within 20%.
$r_{min}$	0.1	Ensures that even in high-density flower clusters, at least a certain number of flowers are retained for subsequent analysis to avoid excessive sparsity.
$r_{max}$	1	For extremely sparse flower clusters, no treatment is required and all are retained.

**Table 3:** Flowering phenotypic traits.

Feature Type	Type Description
Characteristics of flower cluster quantity	Reflect the overall number of flower clusters and the distribution of flower clusters of different sizes, such as the total number of flower clusters and the number of flower clusters of different grades.
Characteristics of flower cluster area	Reflecting the total area of flower clusters and the distribution of different sizes of flower clusters, such as the total area of flower clusters and the total area of different flower clusters.
Characteristics of single flower quantity	Reflect the total number of individual flowers as well as the number of scattered individual flowers and individual flowers within different clusters.
Morphological characteristics of flower clusters	Reflect the shape of flower clusters, such as roundness and compactness.
Spatial distribution characteristics	Reflect the distribution of flower clusters within the tree crown, including the variance of cluster area, dispersion of individual flower positions, and uniformity score.
Characteristics after thinning flowers	The number and area of flower clusters and flowers retained after simulating thinning operations.
Characteristics of flowers within flower clusters	Reflect the density of flowers within a flower cluster, such as the maximum number of flowers in different clusters and the average number of flowers per cluster.

**Table 4:** Mixed_all_opt feature set.

Feature Name	Feature Description	Feature Coefficient
retained_flowers_s2_total	Simulated thinning strategy 2: actual retention of total number of flowers	8.404004536
s2_medium_area	Simulate the total area of type 2 flower clusters in the thinning strategy	−2.730420458
medium_counts	Total number of medium-sized flower clusters directly obtained	2.286744641
s1_supersized_area	The total area size of the super large flower clusters in the simulated thinning strategy one	−1.747947164
s2_supersized_area	The total area size of the super large flower clusters in the second simulated thinning strategy	1.667463577
avg_dynamic_ratio	Simulated thinning strategy—average dynamic retention ratio	−0.922659602
avg_flowers_per_cluster	Average number of flowers per cluster before thinning	0.843435367
s2_dense_area	Simulated thinning strategy 2: total area of dense flower clusters	−0.820126952

**Table 5:** Results of different model training sets.

Model	Feature_Group	*R* ^2^	RMSE (kg)	MAE (kg)	RE (%)
XGBoost [19]	mixed_all	0.978	1.240	0.969	0.097
	mixed_all_opt	0.967	1.516	1.153	0.117
MLP [22]	mixed_all	0.980	1.155	0.901	0.067
	mixed_all_opt	0.951	1.853	1.438	0.125
BPNN [23]	mixed_all	0.929	2.237	1.768	0.172
	mixed_all_opt	0.915	2.452	1.966	0.189
RF [18]	mixed_all	0.980	1.192	0.945	0.087
	mixed_all_opt	0.979	1.207	0.947	0.089
SMR [17]	mixed_all	0.951	1.853	1.441	0.119
	mixed_all_opt	0.933	2.181	1.695	0.143
SVR [21]	mixed_all	0.867	3.048	2.260	0.215
	mixed_all_opt	0.805	3.668	2.790	0.250
LGBM [20]	mixed_all	0.903	2.630	2.147	0.159
	mixed_all_opt	0.873	2.998	2.456	0.188

**Table 6:** Results of different model test sets.

Model	Feature_Group	*R* ^2^	RMSE (kg)	MAE (kg)	RE (%)
XGBoost	mixed_all	0.864	2.998	2.428	0.225
	mixed_all_opt	0.900	2.581	2.043	0.189
MLP	mixed_all	0.746	4.166	3.182	0.292
	mixed_all_opt	0.880	2.678	2.087	0.204
BPNN	mixed_all	0.855	3.062	2.419	0.219
	mixed_all_opt	0.881	2.763	2.214	0.209
RF	mixed_all	0.839	3.270	2.663	0.240
	mixed_all_opt	0.881	2.784	2.265	0.214
SMR	mixed_all	0.835	3.248	2.459	0.221
	mixed_all_opt	0.907	2.408	1.904	0.158
SVR	mixed_all	0.771	3.815	2.864	0.262
	mixed_all_opt	0.793	3.718	2.955	0.266
LGBM	mixed_all	0.753	3.937	3.219	0.225
	mixed_all_opt	0.769	3.788	3.106	0.224

**Table 7:** Training set results for different feature groups.

Model	Feature_Group	*R* ^2^	RMSE (kg)	MAE (kg)	RE (%)
XGBoost	original	0.968	1.484	1.199	0.112
	original_opt	0.961	1.647	1.346	0.129
	strategy1	0.953	1.827	1.461	0.135
	strategy1_opt	0.948	1.915	1.549	0.143
	strategy2	0.971	1.439	1.118	0.115
	strategy2_opt	0.968	1.505	1.173	0.118
	mixed_strategy	0.973	1.387	1.081	0.111
	mixed_strategy_opt	0.966	1.549	1.180	0.117
	mixed_all	0.978	1.240	0.969	0.097
	mixed_all_opt	0.967	1.516	1.153	0.117
	original	0.968	1.484	1.199	0.112
	original_opt	0.961	1.647	1.346	0.129

**Table 8:** Test set results for different feature groups.

Model	Feature_Group	*R* ^2^	RMSE (kg)	MAE (kg)	RE (%)
XGBoost	original	0.832	3.312	2.685	0.233
	original_opt	0.851	3.086	2.533	0.232
	strategy1	0.797	3.680	3.023	0.244
	strategy1_opt	0.800	3.651	2.946	0.251
	strategy2	0.875	2.888	2.318	0.210
	strategy2_opt	0.892	2.693	2.127	0.199
	mixed_strategy	0.856	3.098	2.456	0.216
	mixed_strategy_opt	0.899	2.588	2.039	0.190
	mixed_all	0.864	2.998	2.428	0.225
	mixed_all_opt	0.900	2.581	2.043	0.189
	original	0.832	3.312	2.685	0.233
	original_opt	0.851	3.086	2.533	0.232

## Data Availability

The datasets generated during and/or analyzed during the current study are available from the corresponding author on reasonable request. The data are not publicly available due to restrictions related to cooperative orchard agreements and privacy considerations regarding orchard location and production information.
